# Weight Perception and Lifestyle Awareness in Children and Adolescents: Insights from a Cross-Sectional Study

**DOI:** 10.3390/nu18071017

**Published:** 2026-03-24

**Authors:** Cinzia Franchini, Elena Bertolotti, Beatrice Biasini, Chiara De Panfilis, Susanna Esposito, Alice Rosi, Francesca Scazzina

**Affiliations:** 1 Human Nutrition Unit, Department of Food and Drug, University of Parma, 39 Via Volturno, 43125 Parma, Italyfrancesca.scazzina@unipr.it (F.S.); 2 Unit of Neuroscience, Department of Medicine and Surgery, University of Parma, 43126 Parma, Italy; 3 Pediatric Clinic, Parma University Hospital, Department of Medicine and Surgery, University of Parma, Viale Gramsci 14, 43126 Parma, Italy; 4 Giocampus Scientific Committee, 38 Parco Area delle Scienze, 43124 Parma, Italy

**Keywords:** body image, self-perception, BMI, lifestyle, food habits, KIDMED, physical activity, sleep quality, camps

## Abstract

**Background**: Misperception of body weight has been found to negatively impact both diet and physical activity levels, particularly in youth with overweight and obesity. **Objectives**: This study assessed consistency between actual and perceived weight status and lifestyle factors in a sample of 455 children and adolescents (55% males, 8–13 years) attending a summer camp in Northern Italy. **Methods**: Weight status was defined applying Body Mass Index (BMI) cut-offs. Adherence to the Mediterranean Diet (MD), physical activity level, sleep duration, and sleep quality were assessed through validated questionnaires. Self-perception was evaluated through 5-point Likert scales, with graphical representations. **Results**: Comparison between self-perceived and assessed parameters revealed a poor concordance across all types of variables. Approximately half of participants (43–55%) correctly rated their weight status (κ = 0.12; 95% CI: 0.05–0.19), diet quality (κ = 0.09; 95% CI: 0.02–0.15), physical activity level (κ = 0.18; 95% CI: 0.11–0.26), sleep time (κ = 0.10; 95% CI: 0.03–0.17), and sleep quality (κ = 0.18; 95% CI: 0.12–0.24). Participants 12–13 years old were more likely to have a greater weight status perception compared to younger subjects (OR = 2.13; 95% CI: 1.08–4.21). Being in a condition of overweight or obesity significantly decreased the odds of correct weight perception (OR = 0.13; 95% CI: 0.08–0.21). Similarly, subjects with higher adherence to the MD, adequate sleep time, and low sleep quality were more conscious about their diet and sleep patterns. **Conclusions**: Overall, these findings highlight a certain degree of misclassification, especially in subjects who need to improve their lifestyles, highlighting the potential relevance of fostering accurate self-perception during developmental age.

## 1. Introduction

The prevalence of overweight and obesity in young people in European regions has increased nearly threefold over the past decades, representing a significant public health concern [1]. Young people with excessive body weight are at high risk for developing short- and long-term health complications, including an increased likelihood of becoming obese in adulthood [2,3] and having a reduced overall health status.

On this matter, the beneficial effect of the Mediterranean Diet (MD) in preventing obesity has been widely reported in the scientific literature for both adults and pediatric populations [4,5,6,7]. Furthermore, the MD has been largely studied over the past few decades and recognized as a world-renowned model of healthy and sustainable eating [8,9]. Fruits, vegetables, whole grains, legumes, nuts, seeds, and olive oil are the elements that most characterize this benchmark dietary pattern. Although based on a remarkable consumption of plant-based foods, the MD also allows for a moderate to high intake of fish, a moderate consumption of animal products, such as eggs and dairy, and meat, with a preference for poultry and limited consumption of red meat [10].

Besides promoting proper nutrition, regular exercise represents the other pillar of public health interventions [11] Specifically, according to World Health Organization (WHO) recommendations, children and adolescents aged 5–17 years should reduce the amount of time spent in sedentary activities by engaging in moderate-to-vigorous physical activity for an average of 60 min per day over the course of the week, and bone and muscle strengthening exercises three days per week [12].

In addition, a growing body of literature has highlighted the role of sleep behavior in the overall health status and well-being of children and adolescents [13,14,15], including cognitive functions, physiological mechanisms, emotion regulation, physical growth, and quality of life [16].

As suggested by the WHO Commission on Ending Childhood Obesity report [11], health prevention should be implemented through a multiple strategy based on traditional approaches focusing on healthy nutrition and physical activity, while also giving equal prominence to psychosocial aspects and family support [11]. In this context, a concern to address is represented by body weight misperception (i.e., the difference between an individual’s actual and perceived weight status), which is common among young people [17]. A recent analysis of time trends in 41 countries revealed an overall increase in adolescents’ underestimation of weight status, while a decrease in overestimation was found. Particularly, an increase in correct weight perception emerged only for females. When considering only Italy, the data showed a decrease in correct weight perception and an increase in misperception, both in terms of under- and over-estimation [18]. A further Italian study investigated the role of anthropometric and lifestyle factors in the discrepancy between actual weight status, calculated from anthropometric measurements, and weight status as reported by subjects by answering two questions (i.e., “How much do you weigh?” and “Do you think you are underweight, about the right weight, or overweight?’’). The results of this study suggested that good adherence to the MD reduced the likelihood of misperceiving one’s weight status, both over and under estimation, except for adolescents in underweight. In addition, high levels of physical activity reduced the risk of underestimation in youths with overweight, and the risk of overestimation in females with normal weight [19]. Moreover, previous studies [20,21,22] have emphasized that individuals who misperceived their body image are more likely to adopt behaviors that could lead to malnutrition and poor health outcomes. On this matter, awareness of own weight status is crucial in promoting a change toward a healthy lifestyle and to strengthen the efficacy of the interventions over time [23,24].

With this premise, the present cross-sectional study aimed to provide new insights into the self-perception of children and adolescents. To this end, the actual weight status and lifestyle habits (i.e., diet quality, physical activity, and sleep behavior), respectively obtained through anthropometric measurements and validated questionnaires, were compared with the same parameters self-perceived by the subjects.

## 2. Materials and Methods

### 2.1. Participants and Study Design

This cross-sectional study was conducted over three months (July to September 2021) at the Giocampus summer camp in the city of Parma (North Italy). All children and adolescents between 8 and 14 years old enrolled in the summer camp were invited to participate in the study. Subjects were excluded if they were under the age of 8 or over the age of 14, had metabolic syndromes (hypertension, dyslipidemia, glucose intolerance, or diabetes), were undergoing chronic drug therapy for any pathology (including mental disorders), followed diet therapy, and/or had any previously diagnosed food intolerances or allergies. Recruitment was conducted at the time of registration for the summer camp, during which potential participants and their parents/legal guardians were informed about the purpose and methods of the project. The parents or legal guardians of the subjects provided their written informed consent, while the children and adolescents were asked to give their oral informed assent. The study was conducted in accordance with the ethical standards listed in the Declaration of Helsinki and approved by the Ethical Committee Area Vasta Emilia Nord (AVEN) (Protocol code 28730 on 6 July 2021) and the Giocampus Scientific Board. Each volunteer was identified using an alphanumeric code to guarantee complete anonymity. All data were collected on the same day through the administration of questionnaires by trained researchers from the University of Parma, and the collection of anthropometric measurements by a pediatrician from the “Pietro Barilla Children’s Hospital General and Emergency Pediatrics” of the Azienda Ospedaliera Universitaria di Parma. The questionnaire was divided into 5 sections to collect the following information: (a) Personal information (e.g., date of birth, sex, nationality); (b) Body image and overall lifestyle self-perception; (c) Adherence to the MD; (d) Physical Activity Level; (e) Sleep behavior.

### 2.2. Body Image and Overall Lifestyle Self-Perception

The self-perception of weight status, diet quality, physical activity level, and sleep behavior (i.e., sleep time adequacy and sleep quality) were evaluated using 5-point Likert scales, with graphical representations indicating the different options of body image and lifestyle characteristics. Our questionnaire builds upon the validated body silhouette designed by Collins [25], a recognized approach for assessing self-perception in pediatric population. It should be noted that the original silhouettes, with a 7-point scale, have been modified by removing the most extreme body figures. This adjustment was made upon psychological consultation to prevent potential distress or stigmatization among participants. While the weight status domain was directly adapted from Collins’ silhouettes, we expanded this visual methodology to four additional lifestyle parameters. Each domain was represented by five stylized illustrations designed to reduce cognitive burden and ensure intuitive understanding among young participants, consistent with the principles of cognitive ease. The instrument was refined for this study by a multidisciplinary team of pediatricians, psychologists, and nutritionists to ensure content validity. Its psychometric adequacy was undertaken first through expert review for content validity during the development phase, and then by pilot testing it on a subsample to verify clarity and item comprehensibility.

The volunteers answered the questionnaire by selecting the image or situation that best represented their body image or lifestyle. In particular, the subject would select between extreme conditions such as underweight and obesity, inadequate and adequate diet, sedentary and very active physical activity level, insufficient or excessive sleep time, extreme sleepiness and alertness upon waking up, or situations in between. However, for reasons related to result interpretation, the body perception responses were grouped into three categories. Following the international recoding guidelines of Health Behaviour in School-Aged Children (HBSC) study [26], the responses above normal weight were combined into one single category (“overweight”), responses below normal weight formed another category (“underweight”), while the intermediate category was defined as normal weight. The same criterion was also used to create congruence between the perceived and reported lifestyle variables, identifying three groups for each (diet quality: low, medium, high; physical activity: sedentary or not very active, active, very or extremely active; sleep time: low, adequate, high; sleep quality: low, medium, high).

The percentage of participants who accurately self-classified was calculated for each parameter based on Body Mass Index (BMI) categories, levels of adherence to the MD and physical activity, and categories of sleep time adequacy and sleep quality. The percentage was obtained by dividing the number of subjects who correctly self-classified by the total number of subjects in that specific category. Considering participants who misperceived themselves, the percentage of subjects who underestimated or overestimated their own situation was also evaluated. For instance, normal-weight subjects who perceived themselves as underweight or overweight were categorized as underestimating or overestimating their status, respectively. In addition, the distribution of participants’ self-perception among current categories of weight status and lifestyle variables was also calculated.

### 2.3. Anthropometric Measurements

Anthropometric measurements (i.e., body weight and height) were measured following the WHO guidelines [27] with the volunteer wearing only T-shirt and shorts. Body weight was measured to the nearest 100 g using an electronic scale (MQ919, Maniquick, Niederkassel, Germany). Height was measured to the nearest 100 mm using a portable stadiometer (Leicester Tanita HR 001, Tanita, Arlington Heights, IL, USA). Body Mass Index (BMI) was then calculated as weight in kilograms divided by the square of height in meters and the weight status was determined by applying the International Obesity Task Force (IOTF) BMI cut-offs (kg/m^2^) based on pooled LMS curves [28]. BMI-for-age percentiles were also calculated using the 2000 Centers for Disease Control and Prevention (CDC) growth charts, and weight status was classified according to CDC criteria [29].

### 2.4. Adherence to the Mediterranean Diet

The Mediterranean Diet Quality Index for children and teenagers (KIDMED) questionnaire [30] was used to assess volunteers’ eating habits in terms of adherence to the MD. The KIDMED questionnaire is a validated instrument for children and adolescents widely described in scientific literature, consisting of 16 yes/no questions. Twelve items referred to the consumption of specific foods that are representative of the MD, whereas four items indicated behaviors that are not in accordance with the principles of the MD. A score of +1 or −1 was assigned to positive responses, respectively for MD and non-MD behaviors. The total score ranged between 0 and 12 points, and three levels of adherence to the MD were defined: “high adherence” (score ≥ 8 points), “medium adherence” (score 4–7 points), or “low adherence” (score ≤ 3 points).

### 2.5. Physical Activity Level

The Physical Activity Questionnaire for Older Children (PAQ-C) [31] was applied to estimate the physical activity level of participants. The PAQ-C is a seven-day recall instrument validated for use with school-aged children and adolescents. The questionnaire consisted of 9 items, each of which was scored on a 5-point scale. In addition, a final question was asked to determine whether the described week was representative of a standard week or not. The PAQ-C activity summary score was calculated by averaging the single item scores, and each participant was classified as “sedentary or not very active” (score ≤ 2 points), “active” (score = 3 points), “very active or extremely active” (score ≥ 4 points), or “not usual exercise”. Participants who did not exercise as usual during the week of data collection were excluded from data analysis concerning physical activity.

### 2.6. Sleep Behavior

Sleep behaviors were evaluated by considering both sleep duration and sleep quality. Following the methodology used for previous studies [32,33], sleep duration was calculated by asking participants to report the time they go to bed and wake up, and then calculating the difference. The participants were asked to distinguish between school days and weekend days, and the weighted average of their weekly sleep duration was then calculated considering five school days and two weekend days. Accordingly, sleep time adequacy was classified as “low” (<9 h per night), “recommended” (9–11 h per night), or “high” (>11 h per night), based on the National Sleep Foundation recommendations for children and adolescents [16].

Sleep quality was defined according to rest, fatigue, and mood using the Paediatric Daytime Sleepiness Scale (PDSS) questionnaire [34], consisting of eight multiple-choice questions and validated for use with children and adolescents. A 5-point Likert scale from “Never” to “Always” was used, and each item was scored from 0 to 4 points, except for the third question, whose scale was revers-scored. The final total PDSS score ranges from 0 to 32 points, with higher values corresponding to poorer sleep quality. Specifically, volunteers were classified as having “high sleep quality” (total score ≤ 10 points), “medium sleep quality” (total score 11–20 points), or “low sleep quality” (total score > 20 points).

### 2.7. Statistical Analysis

According to the Kolmogorov-Smirnov test, normality of the sample distribution was rejected, and thus data were expressed as median and interquartile range (IQR) for continuous variables or as absolute number and percentage for categorical variables. Non-parametric Mann-Whitney test and Pearson Chi-square test (χ^2^) were used to investigate possible differences between males and females, for continuous and categorical variables, respectively. The consistency between perceived and assessed parameters was evaluated using the Pearson Chi-square test (χ^2^), while Cohen’s Kappa was calculated to determine the level of agreement beyond chance. Cohen’s kappa values were interpreted according to standard benchmarks considering the following coefficients: ≤0 (poor), 0.01–0.20 (slight), 0.21–0.40 (fair), 0.41–0.60 (moderate), 0.61–0.80 (good), and 0.81–1.00 (optimal). A multistep analytic approach was employed to identify the determinants of correct self-perception. First, Pearson Chi-square (χ^2^) analysis was performed to screen for variables significantly associated with accurate self-perception. Subsequently, these variables were entered into univariate and/or multivariate logistic regression models to identify the main predictors influencing participants’ self-perception. For multivariable models, multicollinearity was assessed using the Variance Inflation Factor (VIF). VIF = 1 indicates no correlation, 1 < VIF < 5 indicates moderate but acceptable collinearity, and VIF > 5 (or >10) signals high multicollinearity requiring corrective measures. For reasons pertaining to the interpretation of Spearman’s rank test results, only for the correlation analysis the PDSS score was reversed. The statistical analyses were performed by the IBM SPSS Statistics for Macintosh, version 29.0 (IBM Corp., Armonk, NY, USA) and the results were considered statistically significant in case of *p* < 0.05.

## 3. Results

### 3.1. Participants’ Characteristics

Out of a total of 455 recruited children and adolescents, three were excluded from the analysis for missing data. The final sample included 452 volunteers with a median age of 9.0 (8.0–11.0) years, mostly males (55%) and with Italian nationality (87%). Anthropometric data and behavioral characteristics of participants and differences between sexes are presented in Table 1.

Most of the participants showed normal weight status, however, one-third of the sample was in overweight or obesity condition. About half of the subjects fell into the category of medium adherence to the MD, whereas 29% and 20% showed high and low adherence, respectively, with a median KIDMED score falling in the medium level of adherence to the MD. Half of the sample led an active lifestyle, whereas the rest of the participants were divided into sedentary or not very active (21%) and very or extremely active (20%). The median sleep time was 9:38 (9:04–10:16), and in most volunteers (71%) the sleep duration was adequate. Reporting the results for weekdays and weekend separately, participants reported longer median sleep duration during weekend days (10:10 vs. 9:30). Finally, the median PDSS score was 13.0 (9.0–17.0) and most participants had a medium (55%) or high (34%) sleep quality.

Overall, no significant differences were found for lifestyle variables between the sexes except for sleep behaviors. Specifically, sleep time was significantly higher in females only when considering the weekend days (*p* = 0.028). This difference did not affect average sleep time, while a significant association was found between sleep time adequacy and sexes (*p* = 0.041). In addition, sleep time adequacy median PSDD score (*p* = 0.010) and distribution among sleep quality categories (*p* = 0.033) differed significantly, showing better sleep quality in males.

### 3.2. Comparison Between Participants’ Self-Perception and Assessed Parameters

The results on the consistency between perceived and assessed variables are presented in Table S1 of the Supplementary Materials. Notably, 75% of subjects with overweight or obesity identify themselves as normal weight; among children and adolescents with low adherence to the MD only 16% are aware of their poor dietary habits, and half of the participants who were found to be sedentary or not very active overestimate their physical activity level. Within the categories of sleep time adequacy and sleep quality, regardless of category, most subjects believe they sleep an adequate number of hours per night and have medium sleep quality. Only 25% and 31% have the self-perception of sleeping too little or too much, respectively, and, among those with low sleep quality, 74% accurately self-classified their condition. Significant associations were found for all the parameters, while the level of agreement tested using Cohen’s Kappa (κ) revealed a poor concordance between two measures (perceived vs. actual) across all types of variables. These findings highlighted the poor ability of children and adolescents to classified themselves correctly. To delve into the possible determinants of correct perception, the share of participants who had properly classified themselves was investigated and the results are shown in Table 2.

**Figure 1:**
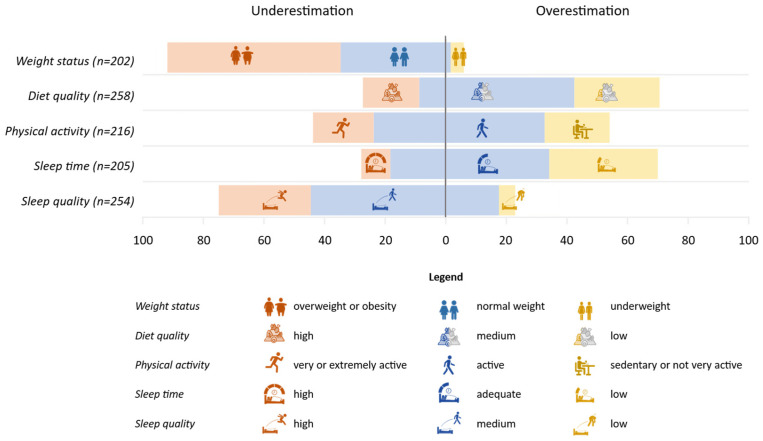
Distribution of participants (%) who misperceived themselves between underestimation or overestimation of their condition reported by different categories of actual weight status (based on IOTF cut-offs), diet quality, physical activity, sleep time, and sleep quality. The percentages of subjects falling within orange, blue, and yellow boxes were calculated on the total sample (shown in brackets) for the different categories of weight status and lifestyle variables.

Overall, approximately half of the sample, ranging from 43% to 55%, correctly rated their weight status, sleep duration, physical activity level, sleep quality, and diet quality in descending order. Considering the distribution of participants with good self-perception among the different subcategories of assessed parameters, significant associations were found for all variables (*p* < 0.001), except for physical activity level. The highest share of subjects with correct self-perception was found among those with a normal weight (74%), a high level of adherence to the MD (63%), an adequate sleep time (67%), and a low sleep quality (74%). Otherwise, considering only the participants who misclassified themselves, Figure 1 reports the distribution of subjects between underestimation or overestimation. Overall, most subjects tended to underestimate their weight status (95%) and sleep quality (64%), whereas diet quality and sleep duration were overestimated by 72% of the subjects in both cases. For physical activity level, the difference between under- and overestimation was less pronounced (45% vs. 55%).

In addition, the bar chart divides the subjects into three categories considering the actual parameters of weight status, diet quality, physical activity, sleep time, and sleep quality. Among the participants who misperceived their weight status (n = 202), 94% underestimated it, of those 58% were in an overweight or obesity condition, while 36% had a normal weight. Among the children and adolescents who misperceived their quality of their diet (n = 258), 72% overestimated it, of those 43% and 29% had medium and low diet quality, respectively. For sleep duration (n = 205), most of the subjects overestimated their sleep duration (71%), of those 35% and 37% had, respectively, a low and medium sleep time based on the National Sleep Foundation recommendation. While considering sleep quality (n = 254), the majority underestimated their sleep quality (76%), of those 31% had a high sleep quality and the others (45%) had a medium sleep quality. Regarding physical activity, participants’ misperception (n = 215) was similar between underestimation (45%) and overestimation (55%).

### 3.3. Determinants of Correct Self-Perception

Besides the significant associations showed in Table 2 (e.g., level of adherence to the MD and correct perception of personal diet quality), other variables related to socio-demographic data were also considered (i.e., age, sex, nationality) and the percentage of subjects with correct weight and lifestyle self-perception was investigated among these categories (Table S2). Significant associations were found only between correct perception of weight status and age (X^2^ = 6.268; *df*: 2; *p* = 0.044). The impact of age on the level of awareness of weight status was also confirmed by univariate and multivariate logistic regression performed (Table 3). Specifically, participants being 12–13 years old were more likely to have a higher level of awareness compared to younger subjects. Likewise, being in a condition of overweight or obesity significantly decreased the odds of correct weight perception. Multicollinearity was assessed using the VIF for significant covariates in the multivariate model (i.e., BMI category and age group), which resulted equal to 1 indicating no significant redundancy among the included predictors. Further analyses were conducted to explore the potential impact of diet and lifestyle factors on perceived parameters (Table S2) (e.g., level of adherence to MD and correct perception of weight status), but no significant associations were found except for correct perception of weight status and sleep time adequacy (X^2^ = 6.491; *df*: 2; *p* = 0.039). In this connection, univariate logistic regression (Table 4) was run for different dependent variables considering the significant associations also previously reported in Table S2 (i.e., self-perceived diet quality, sleep time adequacy, and sleep quality), while sleep time adequacy was also used as covariate in the univariate and multivariate model for predicting correct weight status perception (Table 3). As shown in Table 4, higher adherence to the MD was strongly and positively associated with accurate dietary habits perception, with the highest adherence tertile showing the greatest odds of correct self-perception. Similarly, individuals who reported adequate sleep duration were more likely to correctly perceive their sleep habits than peers in the low- or high-sleeping categories. In contrast, an inverse relationship was noted for sleep quality, confirming that participants reporting low sleep quality showed more awareness of their sleep pattern. These results suggest that adherence to sleep guidelines is associated with a more accurate self-assessment of sleep duration, while poor sleep quality may enhance symptomatic awareness, resulting in superior self-reporting accuracy.

As age emerged as the main determinant of accurate weigh status self-perception, a sensitivity analysis comparing different age groups was performed. The results on the agreement between perceived and measured weight status are presented in Table S3 of the Supplementary Materials. Despite significant associations were found for all age groups (*p* < 0.001), the accuracy of self-perception (%) and level of concordance (κ) increased with age (i.e., 8–9 y: 53%, κ = 0.04; 10–11 y: 55%, κ = 0.14; 12–13 y: 70%; κ = 0.22). These findings corroborated the age as a predictor of self-body awareness.

## 4. Discussion

The present cross-sectional study provides new insights into the self-perception of weight status and lifestyle in a sample of children and adolescents attending a summer camp in Parma (North Italy). Our findings corroborate the notion that school age represents a pivotal period during which fostering body awareness is vital for promoting youth mental and physical health [35]. To this regard, it is important to consider misperception as a multidimensional phenomenon rather than a mere descriptive error, arising from the complex interplay between individual health behaviors and external sociocultural influences [36]. This framework is particularly important in the contemporary context given the massive use of social media, even among the youngest population groups. In particular, such digital environments often foster an increase in body dissatisfaction and distort self-perception through constant upward social comparison, further complicating the alignment between actual and perceived weight status [37,38]. In this regard, several studies have explored the association between weight status and perceived body image, emphasizing better self-classification among children and adolescents with normal weight in different countries, including Italy [19,35,39]. As reported in the literature, the results of the present study also showed a higher prevalence of body weight misperception among participants in an overweight state or living with obesity [19,35,40].

Our results also indicated a positive trend in body self-perception associated with increasing age. This contrasts with a recent longitudinal study by Gualdi-Russo et al. [41], which found no significant changes in body image perception over a three-year period in a sample of Italian adolescents. This discrepancy may be due to the different age ranges investigated, as the latter study focused exclusively on an adolescent population being 11 years old and older.

To the best of our knowledge, no previous studies have investigated the congruence between self-perceived and actual eating habits in Italian children and adolescents. However, similar to the results obtained in this study, a recent study conducted in a sample of Spanish university students showed an overestimation of diet quality in participants with low MD scores [42]. When considering the congruence between reported and perceived levels of physical activity, some studies have indicated that generally youths perceive their physical activity correctly, notably showing better self-perception in subjects with high levels of physical activity [43,44,45]. However, the findings of the present study are consistent with an Italian study which showed a high level of misperception among adolescents leading to an overestimation of their actual physical activity levels [46]. In addition, previous studies [19,35,41,43,47] have indicated that healthy dietary habits and physical activity level exert a positive influence on body perception. However, no significant associations were found in this regard in the present sample. As for the consistency between reported and self-perceived sleep behavior, our findings are in line with those of an earlier study which compared actigraphic sleep recordings and sleep diaries [48], showing that children with high sleep efficiency, indicative of better total sleep time, demonstrated a higher level of accuracy in correctly identifying their sleep habits.

When comparing the weight status and lifestyle habits of our sample with existing literature on similar populations, our results revealed a higher percentage of participants with overweight or obesity compared to the most recent national surveillance reports for children aged 8–9 years in 2023 [49] and adolescents aged 11–13 years in 2022 [50,51] at both regional and national levels. At the local level, previous studies conducted on similar populations of children and adolescents [32,33] reported findings that indicated better weight status. In 2016, 27% of 690 primary school children were found to be in a condition of overweight or obesity [33], whereas only 14% of a sample of 409 high school adolescents was found to be overweight in 2018 [32]. Unfortunately, the data of the present study are in line with a systematic review that reported a worldwide escalation in the prevalence of overweight and obesity among children and adolescents since the onset of the Coronavirus disease (COVID-19) pandemic [52]. As outlined by the authors, a general worsening in the lifestyle habits of young populations was observed, with poorer eating habits and lower levels of physical activity [52] leading to a gradual shift away from the Mediterranean lifestyle in young populations of the Mediterranean basin, including Italy [53,54,55]. Considering the studies that have analyzed the dietary habits of the Italian youth population with respect to the MD over the past decade, it can be observed that the majority of these studies have focused on the eating behaviors of adolescents rather than children, indicating a general low to medium adherence to the MD with a very low percentage of subjects (ranging from 9 to 16%) reporting a high adherence [19,56,57,58,59]. In addition, adherence levels were found to be lower in the southern regions [19,57,59] than in the North [60]. As reported by Archero and colleagues [60], 19% of children and adolescents (6 to 16 years old) living in Novara (Northern Italy) had a high adherence to the MD, approaching the results of the present study, although lower. The present study’s participants demonstrated a level of adherence to the MD that was consistent with the results of another local study carried out in Parma [61]. Notwithstanding, the latter study calculated adherence to the MD using secondary data from 3-day food diaries. Consequently, a direct comparison between the two studies’ results is prevented.

Regarding the physical activity level, the results of the present study are in line with those of a local study conducted on adolescents [32] but not with those of a previous investigation performed on children [33], in which a higher percentage of participants were classified as very or extremely active. This negative trend may be a consequence of the restrictions imposed during the Coronavirus pandemic, as reported by Dallolio and colleagues for a sample of Italian primary school children living in another city in the same geographical region [62]. Keeping in mind the recommendation of performing 60 min of moderate to vigorous physical activity every day [12], only 32% of Italian children being 8 to 9 years old engaged in at least one hour of movement games on five or more days per week in 2019 [63], and around 28% of Italian adolescents being 11 to 13 years old exercised moderate to vigorous physical activity at least 4 days per week in 2022 [49]. However, a comparison of the present results with national data is difficult because of the different instruments used for the assessment of physical activity. Moreover, despite a large body of literature indicating that males are generally more active than females [64,65], no significant association was observed in our results.

Regarding sleep behavior, the participants’ average sleep duration was found to fall within the range recommended by the American Academy of Sleep Medicine for school-aged children (6–13 years old) [16], namely 9–11 h. The results of the present study are consistent with the previously estimated sleep time in children living in Parma [33], whereas a shorter sleep duration was found in a local study conducted on an adolescent cohort [32].

The findings of this study must be evaluated considering its strengths and weaknesses. First, it is necessary to consider the small sample size and the confinement of the study to a single city. In addition, when comparing the weight status and lifestyle habits of our sample with existing literature on similar populations, it should be acknowledged that our study involved a convenience sample of children attending a fee-paying summer camp in a city in Northern Italy. This setting may not fully reflect the broader socio-economic diversity of the general population, as participants are likely to come from families with higher health awareness or a greater predisposition toward physical activity. Consequently, while these findings provide valuable insights, any overall conclusion about the Italian population must be considered with great caution. Additionally, although already noted, it is essential to further emphasize that data collection was conducted in the post-COVID-19 pandemic period. This external factor may have significantly influenced participants’ typical daily routine in terms of food, sleep and physical activity patterns, potentially limiting the generalizability of our findings to typical pre-pandemic lifestyles. On the other hand, the use of validated questionnaires such as KIDMED, PAQ-C, and PDSS represents a strength of the present study. However, it is worth acknowledging that the administration of questionnaires enables evaluation based on self-reported and not objectively measured data. Thus, future studies should include the utilization of activity and sleep trackers to avoid possible bias related to subjectivity and reinforce the results of this pilot study. In addition, the self-perception questionnaire was specifically developed for this study based on relevant literature. Although not previously validated, it was designed to capture the studied aspects of self-perception and its content validity is supported by its design, which was adapted from validated body silhouettes and refined through multidisciplinary expert consultation. However, the single data collection performed in this study precluded an assessment of test-retest reliability. Consequently, the results derived from this tool should be carefully interpreted and further investigations employing a longitudinal design should be undertaken to more comprehensively evaluate the instrument’s stability and reliability over time. Overall, the aforementioned limitations and the cross-sectional design of the study do not allow drawing robust conclusions about children’s and adolescents’ self-perception and its determinants. Therefore, it is advisable to consider these findings as preliminary and to use them as a basis for designing future studies that will allow for a more in-depth examination of the factors that contribute to the accurate self-perception of young people. If future research will confirm the validity of the applied questionnaire and strengthen our findings, this tool could serve as a valuable starting point for developing a screening instrument to be integrated into school-based health programs. In this case, further refinement should adopt a co-design approach by actively involving teachers and students to ensure the development of a questionnaire tailored to the specific needs of the school environment. From a practical point of view, simple and validated self-assessment tools can be incorporated into school-based health programs to enhance awareness among children and adolescents. Examples include silhouette-based scales [25], the KIDMED index [30] for adherence to the Mediterranean diet, the PAQ-C/PAQ-A [31] for physical activity, and the PDSS [34] for sleep habits. Furthermore, Information and Communication Technologies (ICT) may strengthen these strategies by increasing children and adolescent engagement and enabling immediate, personalized feedback. Integrated digital platforms that combine monitoring and health education may increase children’s and adolescents’ perception of their lifestyle habits, and support improvements in their behaviors.

## 5. Conclusions

To the best of the authors’ knowledge, this research contributes to a still limited body of literature evaluating children’s and adolescents’ self-perception across multiple lifestyle domains, rather than focusing exclusively on body image. Notwithstanding, the assessment of children’s and adolescents’ self-perception of their lifestyle is still poorly covered in the existing literature, representing a novel contribution to the field.

Overall, the findings of this study showed a certain degree of misclassification regarding the self-perceived weight status, as well as eating and lifestyle habits. Notably, participants with a normal weight, high adherence to the MD, and adequate sleep time tended to exhibit greater self-classification accuracy compared to their peers with less favorable behaviors. These results may indicate a potential gap in perception among individuals who need to improve their lifestyles. In conclusion, the present study highlights the potential relevance of fostering accurate self-perception during the developmental age. It could be hypothesized that enhancing self-perception might support the effectiveness of future educational health programs and could potentially play a role in addressing the risk of eating disorders and unhealthy development, which are particularly relevant to this population.

## Figures and Tables

**Table 1:** Anthropometric measures and characteristics of participants reported for the total sample and by sex.

Variables	Total (n = 452)	Females (n = 202)	Males (n = 250)	*p* Value
Age (years)	9.0 (8.0–11.0)	9.0 (8.0–10.0)	9.5 (8.0–11.0)	0.244 ^§^
Nationality				0.409 ^†^
Italian	394 (87.2)	179 (88.6)	215 (86.0)	
Non-Italian	58 (12.8)	23 (11.4)	35 (14.0)	
Weight (kg)	36.0 (28.7–44.7)	35.4 (28.3–43.3)	36.5 (29.8–45.3)	0.077 ^§^
Height (cm)	140.1 (132.2–149.0)	138.0 (132.3–148.4)	140.7 (132.1–149.7)	0.212 ^§^
BMI (kg/m^2^)	18.0 (16.2–20.8)	17.7 (15.9–20.3)	18.4 (16.4–20.9)	0.095 ^§^
BMI Category ^δ^				0.054 ^†^
Underweight	18 (4.0)	13 (6.4)	5 (2.0)	
Normal weight	284 (62.8)	125 (61.9)	159 (63.6)	
Overweight/obesity	150 (33.2)	64 (31.7)	86 (34.4)	
BMI-for-age percentiles Category ^Ψ^				0.040 ^†^
<5th percentile	10 (2.2)	7 (3.5)	3 (1.2)	
5th–<85th percentile	298 (65.9)	141 (69.8)	157 (62.8)	
≥85th percentile	144 (31.9)	54 (26.7)	90 (36.0)	
KIDMED score	6.0 (4.0–8.0)	6.0 (4.0–8.0)	6.0 (4.0–8.0)	0.394 ^§^
Adherence to the MD				0.307 ^†^
Low	88 (19.5)	33 (16.3)	55 (22.0)	
Medium	231 (51.1)	106 (52.5)	125 (50.0)	
High	133 (29.4)	63 (31.2)	70 (28.0)	
Physical Activity Level *				0.144 ^†^
Did not exercise as usual	41 (9.1)	23 (11.4)	18 (7.2)	
Sedentary or not very active	94 (20.8)	37 (18.3)	57 (22.8)	
Active	228 (50.4)	108 (53.5)	120 (48.0)	
Very or extremely active	89 (19.7)	34 (16.8)	55 (22.0)	
Sleep duration (hh: mm)				
Week sleep time	9:30 (8:59–10:00)	9:30 (9:00–10:00)	9:20 (8:49–10:00)	0.404 ^§^
Weekend sleep time	10:10 (9:00–11:00)	10:30 (9:30–11:00)	10:00 (9:00–11:00)	0.028 ^§^
Average sleep time	9:38 (9:04–10:16)	9:43 (9:12–10:18)	9:35 (8:55–10:12)	0.103 ^§^
Average sleep time adequacy				0.041 ^†^
Low	101 (22.3)	36 (17.8)	65 (26.0)	
Adequate	322 (71.2)	156 (77.2)	166 (66.4)	
High	29 (6.4)	10 (5.0)	19 (7.6)	
PDSS score	13.0 (9.0–17.0)	13.0 (10.0–17.3)	12.0 (8.0–16.0)	0.010 ^§^
Sleep quality				0.033 ^†^
Low	53 (11.7)	27 (13.4)	26 (10.4)	
Medium	247 (54.6)	120 (59.4)	127 (50.8)	
High	152 (33.6)	55 (27.2)	97 (38.8)	

Data are presented as median (IQR) and absolute number (%) for continuous and categorical variables, respectively. ^§^ Nonparametric Mann–Whitney test for independent sample. ^†^ Pearson Chi-square test. * Analysis was conducted on 411 subjects, excluding participants who did not exercise as usual during the week of data collection. BMI: Body Mass Index; ^δ^ International Obesity Task Force cut-offs; ^Ψ^ Centers for Disease Control criteria; KIDMED: Mediterranean Diet Quality Index in Children and Adolescents; MD: Mediterranean Diet; PDSS: Pediatric Daytime Sleepiness Scale.

**Table 2:** Percentage of participants who were correctly classified in categories of weight status, adherence to the Mediterranean Diet, physical activity level, sleep time adequacy and quality according to their self-perception.

Variables	Total n	Correct Perception n (%)	*p* Value
Weigh status ^δ^			
Total	452	250 (55.3)	<0.001
Underweight	18	9 (50.0)	
Normal weight	284	209 (73.6)	
Overweight/obesity	150	32 (21.3)	
Adherence to the MD			
Total	452	194 (42.9)	<0.001
Low	88	14 (15.9)	
Medium	231	96 (41.6)	
High	133	84 (63.2)	
Physical Activity Level *			
Total	411	196 (47.7)	0.641
Sedentary or not very active	94	47 (50.0)	
Active	228	104 (45.6)	
Very or extremely active	89	45 (50.6)	
Average sleep time adequacy			
Total	452	247 (54.6)	<0.001
Low	104	26 (25.7)	
Adequate	319	212 (66.8)	
High	29	9 (31.0)	
Sleep quality			
Total	452	198 (43.8)	<0.001
Low	53	39 (73.6)	
Medium	247	86 (34.8)	
High	152	73 (48.0)	

Pearson Chi-square test. ^δ^ International Obesity Task Force cut-offs. * Analysis was conducted on 411 subjects, excluding participants who did not exercise as usual during the week of data collection. MD: Mediterranean Diet.

**Table 3:** Logistic regression analyses for having a correct self-perception of weight status (dependent variable).

Variables	Univariate OR (95% CI)	*p* Value	Multivariate OR (95% CI)	*p* Value
Correct self-perceived weight status				
BMI Category ^δ^				
Normal weight	-1-		-1-	
Underweight	0.419 (0.118–1.484)	0.178	0.489 (0.137–1.751)	0.272
Overweight/obesity	0.135 (0.085–0.212)	<0.001	0.132 (0.083–0.209)	<0.001
Age groups				
8–9 years	-1-		-1-	
10–11 years	1.025 (0.681–1.543)	0.906	1.147 (0.722–1.821)	0.561
12–13 years	2.077 (1.146–3.765)	0.016	2.134 (1.081–4.211)	0.029
Average sleep time adequacy				
Low	-1-		-1-	
Adequate	0.564 (0.355–0.898)	0.016	0.628 (0.372–1.062)	0.083
High	0.868 (0.369–2.040)	0.745	1.052 (0.397–2.784)	0.919

^δ^ International Obesity Task Force cut-offs.

**Table 4:** Univariate logistic regression analysis for having a correct self-perception (dependent variables) of diet quality, sleep time adequacy, and sleep quality.

Variables	Univariate OR (95% CI)	*p* Value
Correct self-perceived diet quality		
Adherence to the MD		
Low	-1-	
Medium	3.759 (2.005–7.046)	<0.001
High	9.061 (4.631–17.728)	<0.001
Correct self-perceived sleep time adequacy		
Average sleep time adequacy		
Low	-1-	
Adequate	5.559 (3.365–9.184)	<0.001
High	1.298 (0.525–3.207)	0.572
Correct self-perceived sleep quality		
Sleep quality		
Low	-1-	
Medium	0.192 (0.099–0.373)	<0.001
High	0.332 (0.167–0.660)	0.002

## Data Availability

Data will be made available on request.

## Supplementary Materials

The following supporting information can be downloaded at: https://www.mdpi.com/article/10.3390/nu18071017/s1, Table S1: Participants’ self-perceptions reported by current categories of weight status and lifestyle variables. Table S2: Percentage of participants who correctly classified their weight status, diet quality, physical activity level, sleep time adequacy and quality according to specific socio-demographic data, weight status, and lifestyle parameters. Table S3: Participants’ weight status self-perceptions reported by actual categories of weight status and age groups.

## Abbreviations

The following abbreviations are used in this manuscript:

MD	Mediterranean Diet
WHO	World Health Organization
HBSC	Health Behaviour in School-Aged Children
BMI	Body Mass Index
KIDMED	Mediterranean Diet Quality Index for children and teenagers
PAQ-C	Physical Activity Questionnaire for Older Children
PDSS	Paediatric Daytime Sleepiness Scale
IOTF	International Obesity Task Force
CDC	Centers for Disease Control
