# Supplementary material for: Weight Perception and Lifestyle Awareness in Children and Adolescents: Insights from a Cross-Sectional Study
Source: Nutrients. 2026 Mar 24;18(7):1017. doi: 10.3390/nu18071017 (PMC13074309; doi:10.3390/nu18071017)
Supplement: Supplementary file 1 [file nutrients-18-01017-s001.zip › nutrients-4168304-supplementary.pdf]

Table S1. Participants' self-perceptions reported by actual categories of weight status and lifestyle variables.

	Self-perceived weight status				
	Underweight (n=87)	Normal weight (n=330)	Overweight/ obese (n=35)	κ (95% CI)	p Value (χ^2) †
Weigh status δ				0.12*** (0.05-0.19)	< 0.001
Underweight	9 (50.0)	9 (50.0)	0 (0)		
Normal weight	72 (25.4)	209 (73.6)	3 (1.1)		
Overweight/obese	6 (4.0)	112 (74.7)	32 (21.3)		
	**Self-perceived diet quality**				
	Low (n=40)	Medium (n=188)	High (n=224)		
Adherence to MD				0.09* (0.02-0.15)	< 0.001
Low	14 (15.9)	46 (52.3)	28 (31.8)		
Medium	23 (10.0)	96 (41.6)	112 (48.5)		
High	3 (2.3)	46 (34.6)	84 (63.2)		
	**Self-perceived physical activity level**				
	Sedentary or not very active (n=114)	Active (n=171)	Very or extremely active (n=126)		
Physical Activity Level*				0.18*** (0.11-0.26)	< 0.001
Sedentary or not very active	47 (50.0)	38 (40.4)	9 (9.6)		
Active	52 (22.8)	104 (45.6)	72 (31.6)		
Very or extremely active	15 (16.9)	29 (32.6)	45 (50.6)		
	**Self-perceived sleep time adequacy**				
	Low (n=68)	Adequate (n=288)	High (n=96)		
Average sleep time adequacy				0.10** (0.03-0.17)	0.009
Low	26 (25.7)	58 (57.4)	17 (16.8)		
Adequate	38 (11.8)	214 (66.5)	70 (21.7)		
High	4 (13.8)	16 (55.2)	9 (31.0)		
	**Self-perceived sleep quality**				
	Low (n=68)	Medium (n=288)	High (n=96)		
Sleep quality				0.18*** (0.12-0.24)	<0.001
Low	39 (73.6)	5 (9.4)	9 (17.0)		
Medium	115 (46.6)	86 (34.8)	46 (18.6)		
High	27 (17.8)	52 (34.2)	73 (48.0)		

Data are presented as absolute number (%).δ International Obesity Task Force cut-offs * Analysis was conducted on 411 subjects. excluding participants who did not exercise as usual during the week of data collection. MD: Mediterranean Diet. Cohen's Kappa (κ) *** p<.001; ** p<.01; * p<.05. † Pearson Chi-square (χ^2).

Table S2. Percentage of participants who correctly classified their weight status, diet quality, physical activity level, sleep time adequacy and quality according to specific socio-demographic data, weight status, and lifestyle parameters.

	Weight status		Diet quality		Physical activity		Average sleep time adequacy		Sleep quality	
	**Correct perception n (%)**	**p Value**	**Correct perception n (%)**	**p Value**	**Correct perception n (%)**	**p Value**	**Correct perception n (%)**	**p Value**	**Correct perception n (%)**	**p Value**
**Age group**		0.044		0.607		0.746		0.064		0.286
8-9 years	126 (50.4)		98 (50.5)		106 (54.1)		142 (57.5)		101 (51.0)	
10-11 years	80 (32.0)		66 (34.0)		64 (32.7)		77 (31.2)		73 (36.9)	
12-13 years	44 (17.6)		30 (15.5)		26 (13.3)		28 (11.3)		24 (12.1)	
**Sex**		0.232		0.163		0.942		0.286		0.152
Males	118 (47.2)		94 (48.5)		85 (43.4)		116 (47.0)		96 (48.5)	
Females	132 (52.8)		100 (51.6)		111 (56.7)		131 (53.0)		102 (51.5)	
**Nationality**		0.085		0.147		0.935		0.515		0.308
Italian	224 (89.6)		164 (84.5)		171 (87.3)		213 (86.2)		169 (85.4)	
Non-Italian	26 (10.4)		30 (15.5)		25 (12.8)		34 (13.8)		29 (14.7)	
**Weigh status$^{\delta}$**		<0.001		0.216		0.321		0.574		0.252
Underweight	9 (3.6)		2 (1.0)		5 (2.6)		7 (2.8)		5 (2.5)	
Normal weight	209 (83.6)		134 (69.1)		124 (63.2)		160 (64.8)		138 (69.7)	
Overweight/obese	32 (12.8)		58 (29.9)		67 (34.2)		80 (32.4)		55 (27.8)	
**Adherence to MD**		0.951		<0.001		0.356		0.839		0.082
Low	50 (20.0)		14 (7.2)		44 (22.5)		48 (19.4)		35 (17.7)	
Medium	127 (50.8)		96 (49.5)		93 (47.5)		129 (52.2)		94 (47.5)	
High	73 (29.2)		84 (43.3)		59 (30.1)		70 (28.3)		69 (34.9)	
**Physical Activity Level***		0.852		0.066		0.641		0.149		0.542
Sedentary or not very active	55 (23.7)		39 (22.3)		47 (24.0)		43 (19.2)		38 (21.2)	
Active	126 (54.3)		107 (61.1)		104 (53.1)		131 (58.5)		98 (54.8)	
Very or extremely active	51 (22.0)		29 (16.6)		45 (23.0)		50 (22.3)		43 (24.0)	
**Average sleep time adequacy**		0.039		0.326		0.868		<0.001		0.327
Low	66 (26.4)		37 (19.1)		44 (22.5)		26 (10.5)		47 (23.7)	
Adequate	166 (66.4)		145 (74.7)		140 (71.4)		212 (85.8)		142 (71.7)	
High	18 (7.2)		12 (6.2)		12 (6.1)		9 (3.6)		9 (4.6)	

**Sleep quality**		0.474		0.261		0.884		0.334		<0.001
Low	30 (12.0)		23 (11.9)		23 (11.7)		25 (10.1)		39 (19.7)	
Medium	142 (56.8)		98 (50.5)		106 (54.1)		133 (53.9)		86 (43.4)	
High	78 (31.2)		73 (37.6)		67 (34.2)		89 (36.0)		73 (36.9)	

Pearson Chi-square test. δ International Obesity Task Force cut-offs. * Analysis was conducted on 411 subjects excluding participants who did not exercise as usual during the week of data collection. MD: Mediterranean Diet.

Table S3. Participants' weight status self-perceptions reported by actual categories of weight status and age groups.

	Self-perceived weight status					
	Underweight	Normal weight	Overweight/obese	Correct perception n (%)	κ (95% CI)	p Value (χ^2) †
**Actual weight status** δ						
**8-9 years (n= 239)**				126 (52.7)	0.04 (-0.05-0.12)	<0.001
Underweight	4 (50.0)	4 (50.0)	0 (0.0)			
Normal weight	45 (28.3)	112 (70.4)	2 (1.3)			
Overweight/obese	3 (4.2)	59 (81.9)	10 (13.9)			
**10-11 years (n= 150)**				82 (54.7)	0.14*(0.02-0.25)	<0.001
Underweight	1 (50.0)	1 (50.0)	0 (0.0)			
Normal weight	27 (28.7)	65 (69.1)	2 (2.1)			
Overweight/obese	1 (1.9)	37 (68.5)	16 (29.6)			
**12-13 years (n= 63)**				44 (69.8)	0.22* (0.00-0.44)	<0.001
Underweight	0 (0.0)	0 (0.0)	0 (0.0)			
Normal weight	6 (13.3)	39 (86.7)	0 (0.0)			
Overweight/obese	0 (0.0)	13 (72.2)	5 (27.8)			

Data are presented as absolute number (%).δ International Obesity Task Force cut-offs. Cohen's Kappa (κ) *** p<.001; ** p<.01; * p<.05. † Pearson Chi-square (χ^2).
